# Study on incentive factors and incentive effect differences of teachers in universities and colleges under the view of demographic variables

**DOI:** 10.1186/s40359-023-01426-6

**Published:** 2023-11-08

**Authors:** Danna Hao

**Affiliations:** School of Education Science, Weinan Normal University, Weinan, 714099 China

**Keywords:** Incentive factors, Demographic variables, Teachers in universities and colleges, Differences

## Abstract

The purpose of this study is to explore the factors of University Teachers’ motivation and the differences among the factors under different background variables. Based on a great deal of literatures, this paper classifies the incentive content of teachers in universities and colleges into two aspects: internal incentive and external incentive. Through constructing the incentive structure equation model, this paper analyzes and summarizes the influence factors of the incentive of teachers in universities and colleges from two aspects: internal incentive and external incentive, and finds that external incentive is divided into salary and welfare, organizational environment, career development, and internal incentive is divided into work achievement, individual value, as well as innovation incentive. On this basis, we find that there are significant differences in incentive level based on the characteristics of demographics. Among them, there are significant differences in the factors, including marital status and external incentive. There are significant differences in salary and welfare, organizational environment, work achievement and individual value among different ages. There are significant differences in career development of whether undertaking part-time administrative posts. There are significant differences in salary and welfare, organizational environment and career development among different teaching ages. There are significant differences in organizational environment and career development between different titles. There are significant differences in salary and welfare, organizational environment and incentive between different educational backgrounds, and there are significant differences in innovation incentive between different school types.

## Introduction

At present, in the context of rapid socio-economic development in China and the rapid accumulation of talent resources for university teachers, universities need to stimulate their potential abilities and encourage them to actively engage in scientific research and teaching work, in order to enhance the core competitiveness of universities. At present, various universities in China have introduced various incentive systems to maximize the enthusiasm of teachers in their work and strive to improve their research and teaching performance. However, incentive failures often occur. Therefore, it is particularly important to design a scientific and reasonable incentive system to stimulate teachers’ work efforts. Based on demographic variables, understanding the incentive differences of university teachers under different characteristics can help improve the teaching incentive mechanism for university teachers, enhance their awareness and initiative in teaching, and improve the quality of talent cultivation. It is of great significance for current Chinese universities to achieve the incentive goal of “maximizing the talents of all” in their schools.

### Definition of incentive concept and partition of factors

The term “incentive” is defined in the 7th edition of the Modern Chinese Dictionary with the meaning of stimulating and encouragement by emphasizing the potential incentive to satisfy one’s own individual needs; the English term “arouse” refers to the act or incentive to awaken or motivate a person to something, emphasizing the individual’s response to incentive. The concept in Chinese embodies external factors, while the concept in English reveals internal factors. The Latin word ‘Movere’ means taking action, stimulating, etc. The definition and understanding of motivation vary among different disciplines: motivation is the expression and exploration of emotions [1]. Motivation includes all the procedures involved in the process of initiation, stimulation, development, and termination, mainly reflecting the subjective reflection of the motivated person [2]. Motivation is the behavior taken by organizational members to meet their needs [3]. Motivation is a mediating variable that cannot be directly observed as an intrinsic change [4]. March believes that motivation is a reflection of the process, with the aim of urging members to achieve organizational goals [5]. Incentive is a programmatic process with guiding significance, which is often embodied in stimulating and encouraging, using some means, methods and means to fully explore the embodiment of active participation in the realization of organizational goals [6], whose essence is a kind of means, a kind of behavior embodiment, taking the needs of organizational members as the starting point, adopting many kinds of stimulating ways to guide the activities of organizational members to achieve organizational goals [7]. It is the way used by the members of the organization disgruntled with the present situation, and the behavior which causes the motivator actively to achieve the organizational goal, and in this process, it is divided into internal and external factors [8]. It is the reward of material and spirit, the way to unify the internal goals and organizational goals of the employees [9], and the stimulating process to the psychological incentive, so as to achieve the organizational goal by stimulating the behavior of people and promoting the work of the people [10]. Based on the existing research, this study defines incentive as a means to achieve organizational goals, which is a behavior performance based on meeting the needs of organizational members and achieving organizational goals.

Currently, the academic circle generally divides the incentive into internal incentive and external incentive by using the dichotomy method [11]. There are two kinds of internal needs of internal incentive: the need of ability and the need of self, and external incentive has influence on internal incentive. When external incentive exists, it will reduce its independent ability, thus weakening the effect of internal incentive [12]. When external incentives do not have an impact on internal incentives, and external incentives are added to internal incentives, it actually reduces the existing incentive effect [13]. Cognitive evaluation theory proposes two incentive effects: internal motivation and external motivation [14]. Internal incentive includes achievement, and external incentive includes salary and promotion, etc. [15]. Motivation can be divided into personal factors and environmental factors [16]. The external is the objective environment, and the internal is the subjective factor [17]. For external factors and internal factors, specific internal factors are their own ability and effort, while external factors are luck and objective environment [18]. Qin alvalidated the factors that affect the motivational factors of university teachers through empirical research and established an incentive model. He believed that motivation is a synthesis of various factors, centered on individual needs. When external and internal incentives play a role, the relationship between their variables will also change [19]. The content of incentive is categorized as work itself, salary and welfare, career development, organizational environment, individual value, interpersonal relationship, growth development, work environment, performance evaluation, growth incentive, value incentive, recognition incentive, work incentive, safeguard incentive, as well as environment incentive, etc. [20–24].

To sum up, this study divides external incentive into salary and welfare, organizational environment and career development, and internal incentive into work achievement, individual value and innovation incentive.

## Research design

**Fig. 1:**
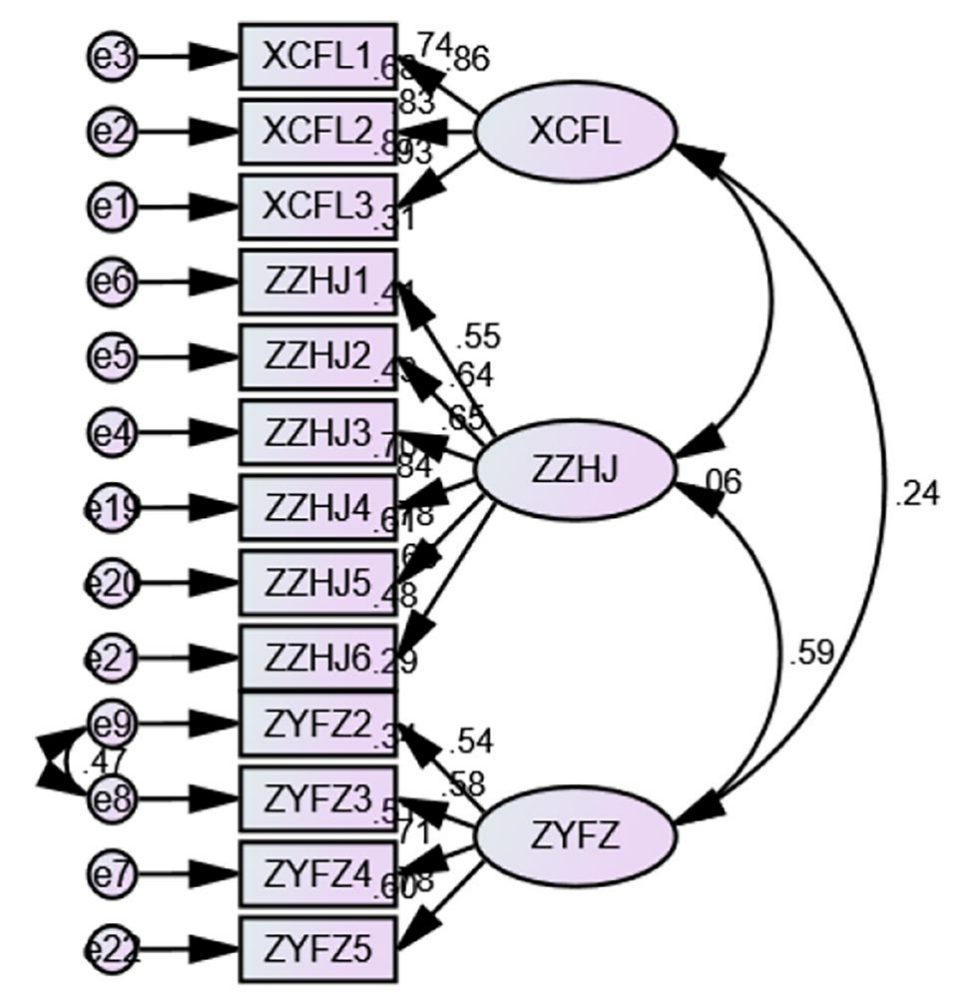
Superimposition and reconstruction of the pre-µCT and post-µCT images of representative samples in each group. From left to right: Obturation material (green), remaining obtruration material post-retreatment (red), superimposed image, occlusal view, occluso-mesial view, and occluso-distal view. **(a)** PTNc, **(b)** RB, **(c) **PTNa, and **(d)** VR.

Draw a research framework diagram based on the research content (Figure 1)

### Questionnaire design of incentive for teachers in universities and colleges

Through literature reading, interview, reference with general scale and so on, the author finally forms the external incentive and internal incentive questionnaire for teachers in universities and colleges, the specific external incentive salary and welfare include: (1) The income will affect my work enthusiasm; (2) The income gap with others will affect my work enthusiasm; (3) The salary will affect my enthusiasm for the job; (4) The more the class hours, the higher the reward; (5) I was paid accordingly for my work. organizational environment include: (1) I can accept all the rules and regulations of the school; (2) I have the opportunity to participate in school decision-making and management; (3) At present, I am satisfied with the school’s teaching facilities, conditions and so on; (4) School management can listen to teachers; (5) I quite agree with the idea of running a school; (6) The school has created a good condition for me to learn and study further。career development include: (1) I am satisfied with the present promotion system; (2) I attach great importance to the promotion of positions and titles; (3) Promotion, and training, etc. can stimulate my enthusiasm for work; (4) There is a chance of promotion through hard work; (5) Promotion is the embodiment of personal development. The specific internal incentive work achievement include: (1) I enjoy the growth brought by my work; (2) I have strong autonomy in my work and can arrange my time reasonably; (3) The new courses and scientific research are challenging and give me incentive; (4) I am loved by my students and respected by my peers; (5) I can arrange the contents of the class according to the actual situation. individual value include: (1) At the moment, my job is what interests me; (2) My work gives expression to my value; (3) I love my job; (4) My work keeps motivating me; (5) I can work for a long time in a row and I enjoy the process. innovation incentive include: (1) I am open to new challenges and new things at work; (2) Solving new problems can make me happy; (3) I will try to solve the dilemma in a new way; (4) I like to bring up new ideas, philosophy and invent new technologies; (5) I enjoy my free play very much.

### Research objects and basic information questionnaire

In this paper, teachers from education and research colleges and universities are taken as the study object. According to Minglong Wu(2009) on the number of samples, generally speaking, more than 200 samples can be called a medium-sized sample. If we want to pursue stable SEM analysis results, the number of samples tested should be 200 or above. Accordingly, given the research objectives, the overall framework of the study, a total of 400 questionnaires was distributed in the ways of field distribution, network distribution, as well as on-site academic conference. Among which, 375 questionnaires were collected, of which 337 were valid questionnaires. By analyzing the basic data of teachers, it is found that the distribution is reasonable and provides a strong data guarantee for further research (see Table 1).

**Table 1:** Distribution List of Performance Incentive Sample Teachers in Colleges and Universities

Demographic variables	classification	number	percentage	Demographic variables	classification	number	percentage
gender	M	131	38.1	Educational background	bachelor’s degree and under bachelor’s degree	52	15.4
	F	206	61.1		master’s degree	215	63.7
age	under 30	54	16		doctor’s degree	70	20.7
	31-35years old	60	17.8				
	36–49 years old	83	24.6	School type	General undergraduate institutions	302	89.6
	41–45 years old	55	16.3		Double first-class university	35	10.3
	46–50 years old	35	10.3	Concurrent administrative position	yes	111	32.9
	over 50	50	14.8		no	226	67
marital status	married	72	21.3	years of teaching	within one year	39	11.5
	unmarried	265	78.6		2–10 years	99	29.3
Professional title	assistant teacher	65	19.2		11–20 years	115	34.1
	lecturer	123	34.6		21–30 years	54	16
	associate professor	103	30.5		over 30 years	30	8.9
	professor	46	13.6				

In this study, for convenience, dimensions are expressed in capital letters, in which salary and welfare—XCFL, organizational environment—ZZHJ, career development—ZYFZ, work achievement—GZCJ, individual value—GRJZ, and innovation incentive—CXJL.

### Exploratory factor analysis of incentive factor

The reliability test is a consideration of the internal consistency and stability of the questionnaire [25]. In general, when Cronbach ’α Alpha is greater than 0.7, the questionnaire only has good reliability. In this study, SPSS23.0 was used to analyze the data of the questionnaire. By measuring the reliability of the questionnaire in 6 dimensions, the overall value of the questionnaire was 0.912, and the salary and welfare was 0.750, the organizational environment was 0.848, the career development was 0.769, the work achievement was 0.771, the individual value was 0.858, and the innovation incentive was 0.843. Cronbach ’α Alpha values exceeded 0.7, indicating that the questionnaire had good reliability.

The KMO and Bartlett’s sphericity test of the questionnaire were measured. The results showed that the KMO value was 0.904. The factor analysis was carried out by the maximum variance method, and the first six factors were extracted when the seventh factor leveled off based on the scree plot. The first six factors were salary and welfare, organizational environment, career development, work achievement, individual value, as well as innovation incentive, with the cumulative variance contribution rate being 61.221%. According to the component matrix after rotation, the factor of salary and welfare 4 less than 0.5 is eliminated (The more the class hours, the higher the reward). salary and welfare 5 (I was paid accordingly for my work), career development 1 (I am satisfied with the present promotion system), work achievement 1 (I enjoy the growth brought by my work), see Table 2.

**Table 2:** Principal Component Analysis of the Incentive Dimensions

Item	XCFL	ZZHJ	ZYFZ	GZCJ	GRJZ	CXJL
XCFL1	0.880					
XCFL2	0.891					
XCFL3	0.916					
ZZHJ1		0.626				
ZZHJ2		0.701				
ZZHJ3		0.720				
ZZHJ4		0.817				
ZZHJ5		0.762				
ZZHJ6		0.743				
ZYFZ2			0.841			
ZYFZ3			0.812			
ZYFZ4			0.531			
ZYFZ5			0.727			
GZCJ2				0.729		
GZCJ3				0.593		
GZCJ4				0.593		
GZCJ5				0.721		
GRJZ1					0.542	
GRJZ2					0.598	
GRJZ3					0.803	
GRJZ4					0.787	
GRJZ5					0.729	
CXJL1						0.668
CXJL2						0.772
CXJL3						0.754
CXJL4						0.723
CXJL5						0.634

Cronbach’s Alpha after deleting the item was tested, the overall Cronbach’s Alpha was 0.899, including salary and welfare 0.904, organizational environment 0.848, career development 0.784, work achievement 0.739, individual value 0.858 and innovation incentive 0.843, which further illustrated that the questionnaire was credible.

The composition reliability, convergence validity and difference validity of AMOS22.0 were analyzed. Composition reliability is an index to measure the consistency of the items in the dimension. It is suggested that the ideal value is greater than 0.5. 0.36 to 0.5 is the acceptable threshold [26]. In this study, CR value is 0.907, 0.849, 0.789, 0.802, 0.868 and 0.848, respectively, and all the indexes are greater than 0.6, showing good consistency of each dimension. In this study, convergent validity was 0.765, 0.489, 0.484, 0.509, 0.570 and 0.531 (see Table 3), respectively, showing good convergence validity among items.

**Table 3:** CR and AVE of incentive factors

variable	item	Unstd	S.E.	T-Value	P	Std	SMC	CR	AVE
XCFL	XCFL3	1.000				0.935	0.874	0.907	0.765
	XCFL2	0.955	0.047	20.456	***	0.828	0.686		
	XCFL1	0.985	0.046	21.644	***	0.857	0.734		
ZZHJ	ZZHJ3	1.000				0.643	0.413	0.849	0.489
	ZZHJ2	1.160	0.117	9.901	***	0.641	0.411		
	ZZHJ1	0.861	0.095	9.026	***	0.561	0.315		
	ZZHJ4	1.487	0.123	12.074	***	0.838	0.702		
	ZZHJ5	1.278	0.107	11.924	***	0.777	0.604		
	ZZHJ6	1.192	0.112	10.655	***	0.699	0.489		
ZYFZ	ZYFZ4	1.000				0.655	0.429	0.789	0.484
	ZYFZ3	0.918	0.106	8.622	***	0.696	0.484		
	ZYFZ2	0.929	0.109	8.544	***	0.671	0.450		
	ZYFZ5	1.130	0.105	10.739	***	0.756	0.572		
GZCJ	GZCJ4	1.000				0.883	0.780	0.802	0.509
	GZCJ3	1.372	0.135	10.164	***	0.708	0.501		
	GZCJ2	1.336	0.146	9.124	***	0.615	0.378		
	GZCJ5	1.166	0.128	9.108	***	0.615	0.378		
GRJZ	GRJZ3	1.000				0.787	0.619	0.868	0.570
	GRJZ2	1.074	0.073	14.745	***	0.782	0.612		
	GRJZ1	1.027	0.075	13.621	***	0.736	0.542		
	GRJZ4	1.067	0.065	16.451	***	0.823	0.677		
	GRJZ5	1.042	0.089	11.704	***	0.632	0.399		
CXJL	CXJL3	1.000				0.835	0.697	0.848	0.531
	CXJL2	0.903	0.065	13.956	***	0.720	0.518		
	CXJL1	0.980	0.072	13.605	***	0.724	0.524		
	CXJL4	1.012	0.066	15.219	***	0.757	0.573		
	CXJL5	0.755	0.069	10.944	***	0.584	0.341		

The discriminant validity was calculated by the way of root opening. Sort was selected under AVE, and then the AVE was calculated by root opening and the root of each ave is larger than that of other related facets [27]. For example, the correlation value of social service is greater than that of other related dimensions,showing that there is difference validity between social service dimension and other dimensions. By analogy, the root values of AVE of education and teaching, organizational dedication, scientific research, innovation incentive, personal value, social service, work achievement, career development and avoidance of loss are0.755、0.713、0.696、0.699、0.875(see Table 4), respectively,all of which were greater than those of other dimensions. So there is there is difference validity between various dimensions given above.

**Table 4:** Discriminant Validity Analyze

	AVE	CXJL	GRJZ	GZCJ	ZYFZ	ZZHJ	XCFL
CXJL	0.531	0.729					
GRJZ	0.570	0.685	0.755				
GZCJ	0.509	0.720	0.619	0.713			
ZYFZ	0.484	0.438	0.470	0.618	0.696		
ZZHJ	0.489	0.373	0.592	0.529	0.502	0.699	
XCFL	0.765	0.042	0.082	0.118	0.275	0.062	0.875

Kline (2010) believed that the distribution of samples in variables would be abnormal if the kurtosis coefficient was greater than 8 and the skewness coefficient of the variable was greater than 3 [26]. Based on the normal distribution results, the observed variables were coincident with the normal distribution (see Table 5).

**Table 5:** Normality of Observed Test

Variable	skew	c.r.	kurtosis	c.r.
CXJL5	-0.513	-3.842	0.976	3.657
CXJL4	-0.457	-3.429	1.239	4.645
GRJZ5	-0.347	-2.601	-0.208	-0.780
GRJZ4	-0.780	-5.847	1.457	5.461
GZCJ5	-0.823	-6.165	1.407	5.274
ZYFZ5	-0.521	-3.908	0.160	0.601
ZZHJ6	-0.580	-4.348	-0.096	− 0.361
ZZHJ5	-0.546	-4.091	0.149	0.559
ZZHJ4	-0.351	-2.628	-0.574	-2.153
CXJL1	-0.707	-5.298	1.674	6.273
CXJL2	-0.787	-5.899	2.169	8.128
CXJL3	-0.688	-5.153	2.561	9.596
GRJZ1	-0.806	-6.042	1.028	3.853
GRJZ2	-0.767	-5.747	1.077	4.034
GRJZ3	-0.844	-6.327	2.011	7.534
GZCJ2	-0.765	-5.736	0.723	2.708
GZCJ3	-0.586	-4.395	0.812	3.044
GZCJ4	-0.455	-3.410	1.727	6.472
ZYFZ2	-0.667	-4.995	0.714	2.677
ZYFZ3	-0.832	-6.235	1.488	5.576
ZYFZ4	-0.595	-4.458	0.106	0.398
ZZHJ1	-0.784	-5.874	0.554	2.076
ZZHJ2	-0.073	-0.546	− 0.510	-1.910
ZZHJ3	-0.513	-3.843	0.152	0.571
XCFL1	-0.851	-6.380	0.351	1.316
XCFL2	-0.717	-5.377	0.015	0.057
XCFL3	-0.910	-6.819	0.573	2.146
Multivariate			322.508	74.805

### Analysis of empirical results

**Fig. 2:**
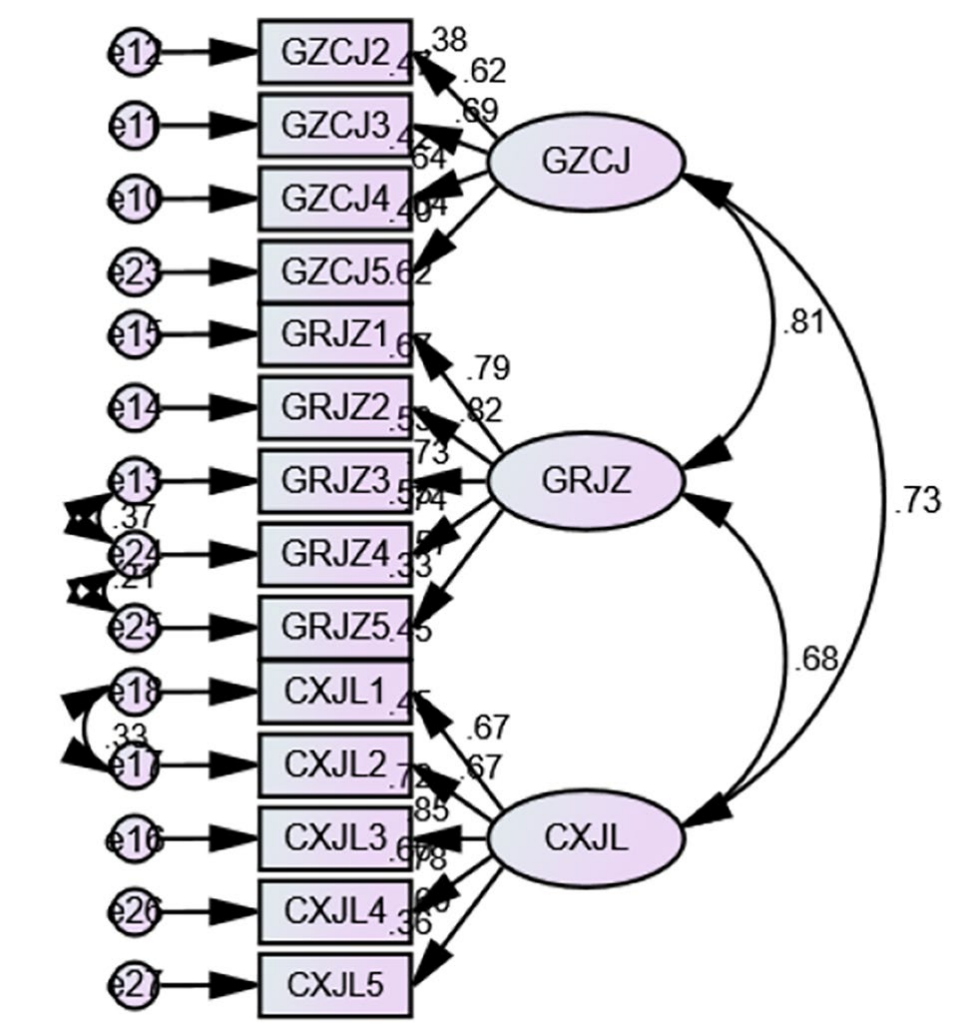
The final model of external incentive for University Teachers

The model fitting degree of structural equation model was analyzed through structural equation model, and the initial model of external incentive of teachers in universities and colleges was formed, among which, the square value was 225.814, the degree of freedom was 62, the ratio of square value to the degree of freedom was 3.642, GFI was 0.905, AGFI was 0.860, NFI was 0.892, IFI was 0.920, TLI was 0.898, CFI was 0.919 and RMSEA was 0.089. Among them, the ratio of chi-square value to the degree of freedom did not meet the standard of less than 3, and RMSEA did not meet the limit of less than 0.08. Therefore, the model is modified according to the correction index. After the correction, the chi-square value was 167.258, the degree of freedom was 61, the ratio of chi-square value to the degree of freedom was 2.742, GFI was 0.929, AGFI was 0.894, NFI was 0.920, IFI was 0.948, TLI was 0.933, CFI was 0.947, and RMSEA was 0.072, and the fitting degree reached the standard (see Fig. 2).

**Fig. 3:**
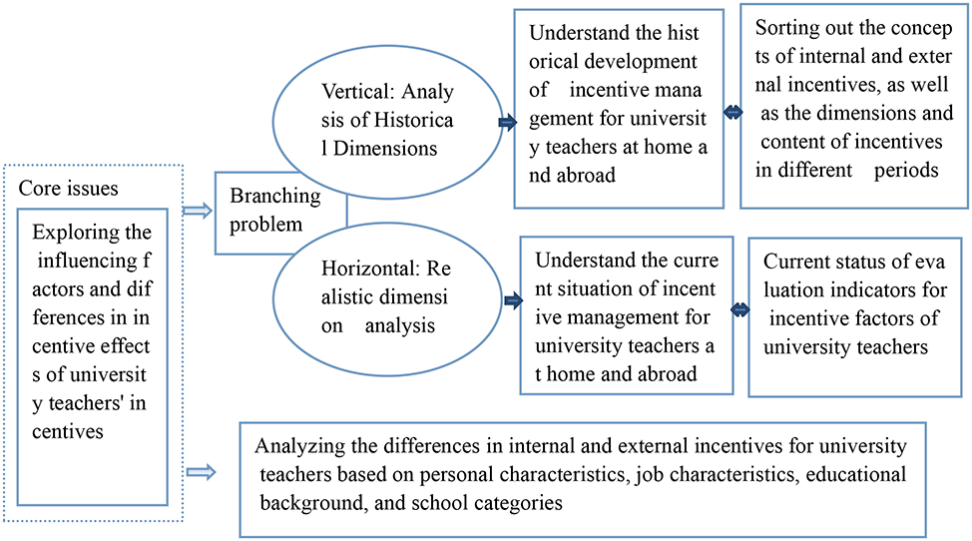
The final model of external motivation for University Teachers

The model fitting degree of structural equation model was analyzed through structural equation model, and the initial model of external incentive of teachers in universities and colleges was formed, among which, the square value was 268.370, the degree of freedom was 74, the ratio of square value to the degree of freedom was 3.627, GFI was 0.882, AGFI was 0.833, NFI was 0.883, IFI was 0.912, TLI was 0.892, CFI was 0.912 and RMSEA was 0.088. Among them, the ratio of chi-square value to the degree of freedom did not meet the standard of less than 3, and RMSEA did not meet the limit of less than 0.08. Therefore, the model is modified according to the correction index. After the correction, the chi-square value was 194.371, the degree of freedom was 71, the ratio of chi-square value to the degree of freedom was 2.738, GFI was 0.918, AGFI was 0.879, NFI was 0.915, IFI was 0.945, TLI was 0.928, CFI was 0.944, and RMSEA was 0.072, and the fitting degree reached the standard (see Fig. 3).

### Verification and conclusion of incentive differences under demographic variables

Based on the characteristics of teachers’ groups, this study explores the significance of incentive factors for teachers in universities and colleges in different backgrounds. Because of the differences in the number and mode of the item design of demographic variables, the main ways of this study are independent sample t-test, single factor variance analysis, and so on. By comparing the different dimensions of incentive factors, the differences among teachers in gender, education and school categories are further analyzed.

(I) Incentive significance analysis based on individual basic characteristics.

1. There were significant differences for marriage status in salary and welfare, organizational environment, career development, work achievement, individual value and innovation incentive, and the incentive of unmarried to salary and welfare was significantly higher than that of married to salary and welfare.
2. As for age comparison, there were significant differences in salary and welfare, organizational environment, work achievement and individual value among different ages. Among them, there were significant differences between 30 years old and 46 ~ 50 years old and 51 years old and above in salary and welfare. The average age of 30 years old and below was significantly higher than that of 46 ~ 50 years old and 51 years old or above, 31 ~ 35 years old, 36 ~ 40 years old and 46 ~ 50 years old, and the average age of 31 ~ 35 years old and 36 ~ 40 years old was significantly higher than that of 46 ~ 50 years old. 30 years old and below and 31 ~ 35 years old, 36 ~ 40 years old, 41 ~ 45 years old, 46 ~ 50 years old had significant difference in terms of organizational environment, and the average age of 30 years old and below was significantly higher than that of 31 ~ 35 years old, 36 ~ 40 years old, 41 ~ 45 years old, 46 ~ 50 years old, and 41 ~ 50 years old and 51 years old and above had significant difference, 36 ~ 40 years old and 51 years old and above had significant difference, and the average age of 51 years old and above was higher than the average age of 36 ~ 40 years old and 41 ~ 50 years old; in terms of work achievement, there were significant differences between 36 ~ 40 years old and 30 years old and below, 31 ~ 35 years old, 41 ~ 45 years old, 46 ~ 50 years old, 50 years old and above, and 41 ~ 45 years old and 36 ~ 40 years old, there were significant differences between 51 years and above and 36 ~ 40 years, and the average age of 30 years old and below and 51 years old and above was significantly higher than that of 31 ~ 35 years old, 36 ~ 40 years old, 41 ~ 45 years old and 46 ~ 50 years old; in terms of individual value, there were significant differences between 36 ~ 40 years old and 30 years old and below, 41 ~ 45 years old and 51 years old and above, and the average age of 30 years old and below and 51 years old and above were significantly higher than those of 31 ~ 35 years old, 36 ~ 40 years old, 41 ~ 45 years old and 46 ~ 50 years old.

(II) Analysis of incentive significance based on job characteristics.

1. Whether part-time administrative posts or not had significant difference in career development, and having part-time administrative posts had more influence on career development incentive than that of having no part-time administrative posts, it shows that teachers with administrative posts preferred to break through themselves in the career development.
2. The comparison of teaching age shows that there were significant differences in salary and welfare, organizational environment, career development and job achievement incentive among different teaching age. Among them, there were significant differences between 2 ~ 10 years and 11 ~ 20 years, 21 ~ 30 years, and 31 years and above, and the average of 2 ~ 10 years was significantly higher than that of other years. In organizational environment, there were significant differences between within 1 year and 2 ~ 10 years, 11 ~ 20 years and 21 ~ 30 years, and the average value within 1 year was significantly higher than that of other years. In terms of career development, there were significant differences between 11 ~ 20 years and within 1 year and 2 ~ 10 years, and the average value of 1 year and within was obviously higher than that of other years; in terms of work achievement, there were significant differences between 11 ~ 20 years and 1 years and within, 2 ~ 10 years, 31 years and above, and the average value of 31 years and above was obviously higher than that of other years, 2 ~ 10 years is more sensitive to salary and welfare incentive, and perhaps it is because that the teachers at this periods were unstable and wanted to improve life through salary and welfare incentive; teachers with teaching experience within 1 year were curious about working environment, so they paid more attention to the campus environment, cultural atmosphere, and so on, at the same time, they strove to participate in various activities to promote themselves through such activities after being employed, in order to adapt to and integrate into the role of teachers faster; teachers who have been teaching for 31 years or more were more sensitive to job achievement incentives, and on the basis of meeting other needs, such teachers wanted to be successful in their positions to prove their self-worth.
3. There were significant differences between different titles, organizational environment and career development in the comparison of titles. In the organizational environment, there were significant differences between teaching assistant and lecturer, associate professor, and the average value of assistant professor was higher than that of other professional titles. In the career development, there were significant differences between assistant professor, teaching assistant and professor, and the average value of professors was higher than that of other professional titles, and maybe professors began to pay attention to higher development after realizing the promotion of professional titles, such as administrative promotion.

(III) Incentive significance analysis based on educational background.

There were significant differences in salary and welfare, and organizational environment incentive between different educational backgrounds. In terms of salary and welfare, there were significant differences between undergraduate and below and master, doctor and above, the average of doctor and above was higher than that of undergraduate and below and master; in terms of organizational environment, there were significant differences between undergraduate and below and master, and the average value of undergraduate and below and master was higher than that of other educational background. In the survey, bachelor’s degree teachers paid more attention to the school environment including cultural atmosphere.

(IV) Incentive significance analysis based on school category.

Different schools had significant differences in innovation incentive, and universities that participate in China’s construction plan of world-class universities and first-class disciplines were significantly higher in innovation incentive than ordinary undergraduate universities, reflecting that universities that participate in China’s construction plan of world-class universities and first-class disciplines were more innovative.

## Discussion and suggestion

By the research, it is found that the university or the competent department should make appropriate adjustments and changes based on the actual characteristics of university teachers when making relevant measures, so as to improve the performance of teachers in universities and colleges through incentive policy.

Based on the analysis of personal characteristics, it is recommended to adopt a "one matter, one discussion" approach based on marital status in response to differences in salary and benefits. When formulating incentive policies, different reward policies should be adopted based on marital status. In response to differences in organizational environment, incentives are given to relatively young teachers based on their age, and more suitable reward methods are given to create a youthful office space. In response to the differences in work achievements and personal values, young teachers have more needs and energy. They have active thinking and strong innovation, and hope to have more opportunities to showcase themselves. They also hope to showcase their value through their own abilities. In terms of incentive policies, they should focus on stimulating young people and helping young teachers, while older teachers have differences in personal values compared to other age groups. In response to this result, In terms of policy formulation, it is recommended to establish a “model promotion” model, encourage older teachers to drive young teachers and form “assistance groups”, so that the elderly can reflect their value through imparting experience and other forms. Based on the analysis of job characteristics, distinguish the incentive methods for teachers who assume administrative positions from those who do not, so that teachers with administrative expertise can develop through the promotion of administrative positions. According to different teaching years, the salary and benefits of teachers who have worked for 2-10 years are more effective, indicating that teachers at this stage may have just entered the workforce, and stable economic income and additional benefits are directly related to their lives. Therefore, when formulating policies, different forms of incentives should be given based on the characteristics of teaching years and their concerns. Encourage young teachers more, provide them with more opportunities for promotion and external communication, and focus on their job growth. Set corresponding incentive methods based on the characteristics displayed by different professional titles. Based on the analysis of educational background, due to historical reasons, most teachers with bachelor’s degrees are older and have a long teaching experience. Therefore, incentive measures for such teachers should be based on the teaching environment, personal respect of teachers, and the school’s overall policy of valuing older teachers. However, promotion incentives for university teachers with master’s and doctoral degrees, as well as those with bachelor’s and below degrees, are more sensitive, Therefore, adopting direct teaching and scientific research performance evaluation is more appropriate. Based on the analysis of school categories, due to the degree of policy inclination, allocation of education funds, quality of student resources, and uneven regional economic development, local universities have weaker incentives for innovation than double first-class universities. Therefore, the innovation incentive policies of local universities must have “local” characteristics, and formulate teacher incentive policies that are suitable for the actual situation of the school based on the location of the school, On the one hand, it is necessary to establish an incentive system for talents to be “retained”, ensuring that effective incentive measures can provide a good teaching and research environment for local university teachers. On the other hand, it is necessary to develop ". On the other hand, it is necessary to establish a “introduced” incentive system to ensure that the school’s talent incentive system maintains strong attractiveness, in order to ensure the stable growth of talent resources in universities.

1. Lay emphasis on the material needs of teachers and pay attention to the needs of teachers’ lives.

In the aspect of salary and welfare, unmarried teachers’ desire for salary is much higher than that of married teachers. Therefore, we should refine the key points according to the needs of teachers at different levels and insist on the combination of material and spirit, so as to put forward corresponding incentive measures when making incentive mechanism for teachers.

2. Scientific and rational construction of teacher incentive and evaluation system.

First of all, incentive policy should be specific and clear, and directly related to the performance of teachers, and at the same time, in terms of the operation, it should be simple and clear; in the specific policy-making, the corresponding policies should be made in accordance with different technical posts, to rationally analyze and accurately position by adhering to the concept of fairness and justice; in addition, the assessment should be diversified, with overall consideration and dynamic design, and it should be adjusted timely according to the changes in the environment and so on, and at the same time, the regular and irregular incentives should be combined to make teachers work hard to achieve their goals.

3. Multiple needs coexist to create more opportunities for achievement.

The demand of teachers with different background information is different. Only teachers with different background and different groups can see the hope and be encouraged, so that the whole teacher team can be full of vitality, especially for teachers with lower educational background, younger age and lower professional title, it should provide greater opportunities for promotion and development for them, which is an important measure to promote teachers’ incentive. At the same time, in the process of adjusting teacher incentive factors, we should pay attention to the importance of smooth transition, so as to avoid the big shock caused by the change.

## Data Availability

The datasets used and/or analyzed during the current study are available from the corresponding author on reasonable request.
